# Endometriosis and its global research architecture: an in-depth density-equalizing mapping analysis

**DOI:** 10.1186/s12905-016-0336-0

**Published:** 2016-09-21

**Authors:** Dörthe Brüggmann, Alexandra Elizabeth-Martinez, Doris Klingelhöfer, David Quarcoo, Jenny M. Jaque, David A. Groneberg

**Affiliations:** 1 Department of Obstetrics and Gynecology, Keck School of Medicine, University of Southern California, 2020 Zonal Ave, IRD 509, Los Angeles, 90089-9300 CA USA; 2 Institute of Occupational Medicine, Social Medicine and Environmental Medicine, Goethe-University, Theodor-Stern Kai 7, 60590 Frankfurt, Germany

**Keywords:** Endometriosis, Density equalizing, Mapping, Research architecture

## Abstract

**Background:**

Endometriosis is one of the most common gynecological diseases. It is still a chameleon in many aspects and urges intense research activities in the fields of diagnosis, therapy and prevention. Despite the need to foster research in this area, no in-depth analysis of the global architecture of endometriosis research exists yet.

**Methods:**

We here used the NewQIS platform to conduct a density equalizing mapping study, using the Web of Science as database with endometriosis related entries between 1900 and 2009. Density equalizing maps of global endometriosis research encompassing country-specific publication activities, and semi-qualitative indices such as country specific citations, citation rates, h-Indices were created.

**Results:**

In total, 11,056 entries related to endometriosis were found. The USA was leading the field with 3705 publications followed by the United Kingdom (952) and Japan (846). Concerning overall citations and country-specific h-Indices, the USA again was the leading nation with 74,592 citations and a modified h-Index of 103, followed by the UK with 15,175 citations (h-Index 57). Regarding the citation rate, Sweden and Belgium were at top positions with rates of 22.46 and 22.26, respectively. Concerning collaborative studies, there was a steep increase in numbers present; analysis of the chronological evolution indicated a strong increase in international collaborations in the past 10 years.

**Conclusions:**

This study is the first analysis that illustrates the global endometriosis research architecture. It shows that endometriosis research is constantly gaining importance but also underlines the need for further efforts and investments to foster research and ultimately improve endometriosis management on a global scale.

**Electronic supplementary material:**

The online version of this article (doi:10.1186/s12905-016-0336-0) contains supplementary material, which is available to authorized users.

## Background

Endometriosis is one of the most common benign gynecological diseases affecting 1 in 10 women of childbearing age; its management constitutes a daily routine in the practice of obstetrician/gynecologists [1–3]. In the United States (USA), it represents the third leading cause of gynecologic hospitalization, limits workforce participation and impacts both a woman’s physical and mental well being [4]. Hence, endometriosis poses a tremendous burden on individual and community health: In the USA alone, the related total societal costs were projected at $70 billion in 2009 [5].

Endometriosis is an estrogen-dependent condition characterized by endometrial glands and stroma located outside the uterine cavity [6–9]. Implants are found in the peritoneum, the ovaries and the rectovaginal septum constituting three different disease entities [10]. Endometriosis is a commonly underdiagnosed condition: Its epidemiologic assessment is hampered by a high number of obscure cases in the general population and the lack of non-invasive diagnostic tools [11, 12]. However, endometriosis is estimated to have a prevalence of up to 5 % in fertile and 25–40 % in infertile women [8]. Additionally to debilitating symptoms such as dysmenorrhea, chronic pelvic pain and infertility, 8–30 % of patients will develop endometriosis-associated ovarian cancer during their lifetime [13–15]. Currently, there is no cure for endometriosis but several treatment protocols exist for the management of associated sequelae [8, 16, 17].

Although substantial progress has been achieved investigating the pathogenesis and pathophysiology of the disease, endometriosis still belongs to the most enigmatic and complex conditions in the field of obstetrics and gynecology (OB/GYN). Further multidisciplinary, translational and clinical research is needed [4, 11, 18] to define disease origins, to develop non-invasive diagnostic tools and in-vitro models aiming to evaluate the process of malignant transition as well as novel treatment approaches [6, 12, 19, 20]. In this respect, it is commonly accepted that further randomized clinical trials, which focus on proposed etiopathogenic mechanisms and therapeutic innovation, are necessary to identify more conclusive, evidence-based answers [8].

Besides posing a scientific challenge, endometriosis constitutes a major burden on female health worldwide. Hence, we deduce that research and public health efforts have to be strengthened to ensure future successes. To plan these research endeavors accordingly so they meet identified shortcomings and to supply decision makers with information concerning funding strategies, it is the objective of the study to assess the present scientific performance in the field. The ‘New Quality and Quantity Indices in Science’ (NewQIS) project [21, 22] combines scientometric tools and advanced density equalizing mapping procedures [23] to depict the global research architecture and to evaluate country-specific productivity in the framework of the scientific landscape. We also aim to guide individual scholarship and the publication of own research by presenting the 10 most cited articles and the most proliferative journals in the field of endometriosis research.

## Methods

### NewQIS protocol

We employed the previously established NewQIS platform to conduct this study [21, 22]. The method encompasses the use of advanced visualization algorithms such as Gastner and Newman’s density equalizing calculations and scientometric tools in order to evaluate and visualize endometriosis-associated research activity [21, 22].

### Data source

As described previously [24, 25], the database Web of Science (WoS, Thomson Scientific) was used for data collection. We based this study on this particular resource because WoS provides the unique opportunity to analyze the global publication activity and to perform an in-depth citation analysis, which includes the calculation of combined semi-qualitative country- and endometriosis-specific indices such as the modified h-indices or average citation rates.

### Search strategy

In the presented approach, we identified endometriosis-specific publications by the use of the specific search term ‘(endometrios*)’. The operator * was applied to identify both singular and plural cases in title, abstract and key words of all eligible publications listed in the WoS.

### Timeframe

The search was performed in 2010; the analyzed timeframe covered the years between 1900 (01-01) and 2009 (31-12). As discussed in previous studies, results from 2010 onwards were not considered due to incomplete data acquisition (i.e. citation rate) in order not to hamper the global citation analyses.

### Data analysis and categorization

All endometriosis-related publications issued in the timeframe of 110 years were analyzed using our standardized protocol and categorized with respect to publication date, country of origin, source title, and authors [26]. Also, the numbers of citations were retrieved for each publication and the average citation per item (CR, citation rate) were calculated as previously described.

After transfer the raw data to excel charts, the findings were illustrated in diagrams and visualized by density-equalizing mapping projections (DEMP) [26]. The current DEMP analysis was based on the algorithm of Gastner and Newman [23], so the territories of the different countries publishing endometriosis research were resized in proportion to our selected variables. In the present context, we used these visualization algorithms to draw a sketch of worldwide endometriosis-related research activities illustrating the global distribution of country-specific numbers of published items and related citation rates.

### Analysis of research cooperations

To investigate endometriosis-related research collaborations between the different countries, the affiliations of the endometriosis-related publications were screened as earlier described for other diseases [24, 26]. If at least two authors, attributed to different countries, contributed to an endometriosis article, this relationship was defined as a collaborative publication.

### Journal analysis

We evaluated journals publishing endometriosis research by analyzing quantitative and qualitative aspects, e.g. number of published endometriosis-specific articles as well as citations these items received (CR).

## Results

### Global endometriosis research activity

In total, 11,056 endometriosis-related publications were identified. These items were published in 20 different languages: 93.0 % were written in English, 3.4 % in German, 2.3 % in French, and 0.5 % in Spanish, respectively.

**Fig. 1:**
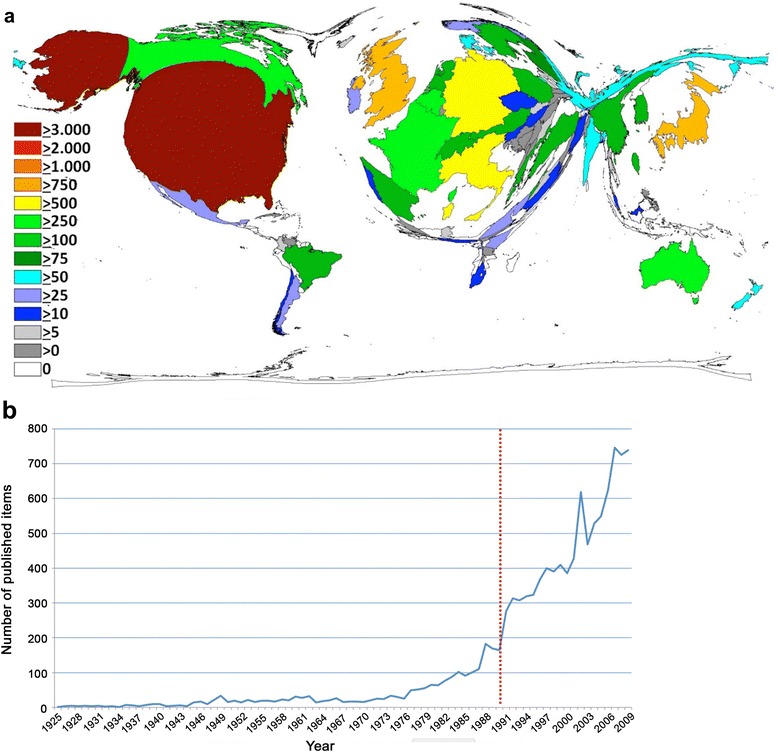
**a** Density-equalizing map of total endometriosis publications in each country. The area of each country was scaled in proportion to its total number of publications. Different colors encode numbers of endometriosis publications. This DEMP figure was generated by the authors as described in the methods section. **b** Chronological evolution of endometriosis-related publications. The red dotted line indicates the steep increase in publication activity visible after 1990

Out of 11,056 publications, 10,881 were attributed to one or more originating countries and issued by authors from 88 nations. The USA was most productive with 3705 publications (p). It was followed by the United Kingdom (UK, p: 952), Japan (p: 846), Italy (p: 720), Germany (p: 715), France (p: 497), Belgium (p: 378), Canada (p: 375), Australia (p: 270) and Spain (p: 213). In the DEMP-analysis (Fig. 1a), major parts of Asia including Russia and China (with the exception of Japan), Africa and South America (except Brazil), appear minimized. The DEMP visualizes that North America clearly constitutes the scientific center of endometriosis research, followed by Western Europe.

In the analysis of the chronological evolution of endometriosis research (Fig. 1b), a significant increase in annual publication numbers was visible from 1980 onwards. The steep increase after 1990 can be explained by the fact that the WoS started to include abstracts of the publications in the database, which increased the likelihood to identify endometriosis-related items by topic search. Correspondingly, a larger number of publications and related citations could be identified in our search. The years with the highest publication activities included 2007 (746 annual publications), 2009 (739 annual publications) and 2008 (726 annual publications). The highest growth in publication output was present between 2002 and 2003 with an increase from 428 to 618 %. In the last 10 years of our analysis spanning 110 years of endometriosis research, 6221 publications were published (equals 56 % of all).

### Citation analysis

**Fig. 2:**
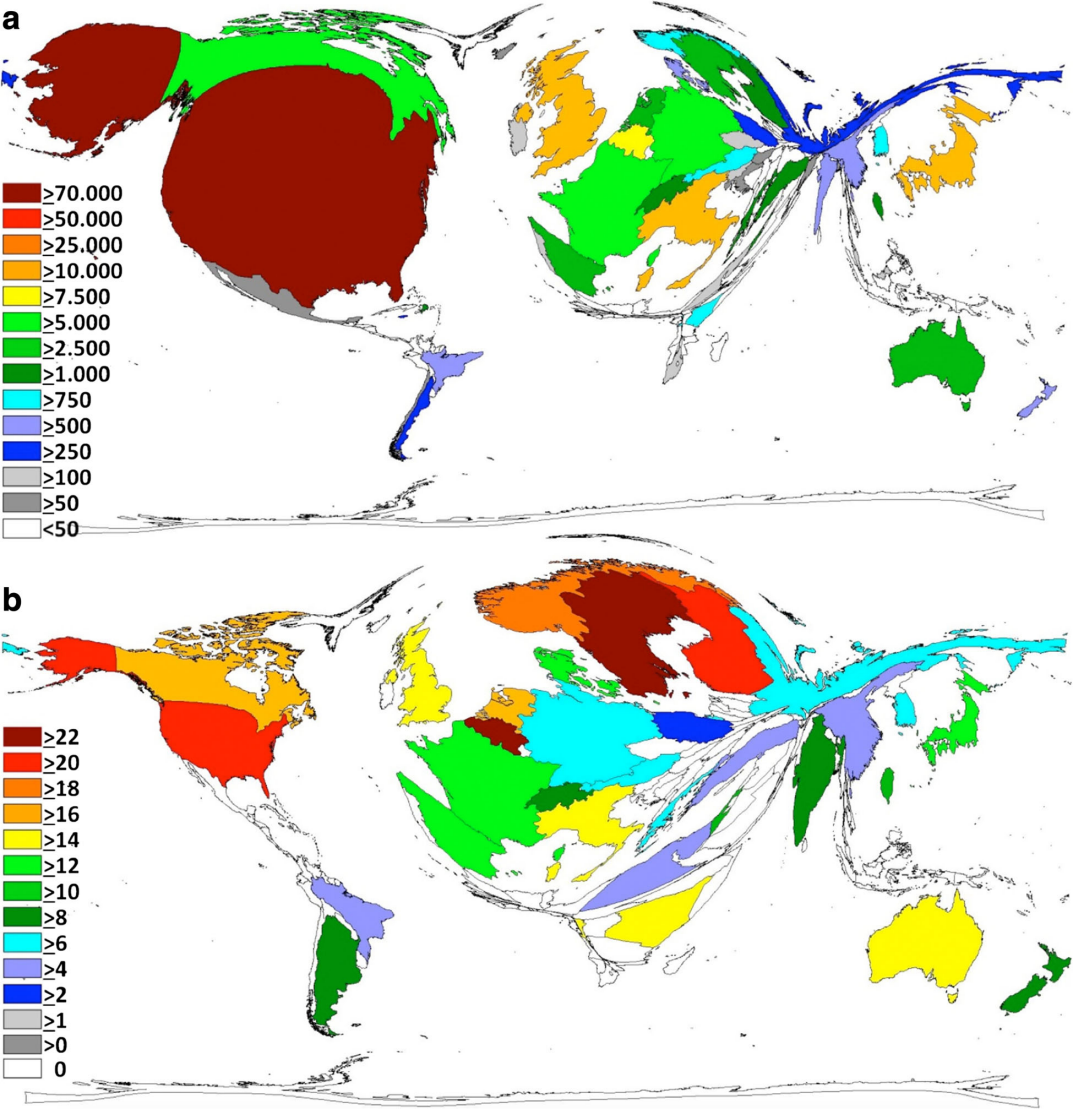
**a** Density-equalizing map of country total citations of endometriosis publications. The area of each country was scaled in proportion to its total number of citations. Colors encode numbers of citations. **b** Density-equalizing map of country citation rate of endometriosis publications. The area of each country was scaled in proportion to its citation rate. Colors encode numbers of citations. These DEMP figures were generated by the authors as described in the methods section

In the country-specific citation analysis (Fig. 2a), again, the USA was found to be the leading nation with a total of 74,592 citations, followed by the UK with 15,175 citations, Japan with 11,704 citations and Italy with 10,147 citations. DEMP results of total citations resembled the aforementioned DEMP analysis of total publications numbers with some minor exceptions, i.e. Belgium was ranked 5^th^ with 8489 citations (Fig. 2a). In Additional file 1, we compiled the 10 most cited articles in the field.

The calculation of the country-specific citation rate (citations per endometriosis publication of a country, with a threshold of 30 publications per country) was used as a semi-qualitative measure (Fig. 2b). It pointed to a high average citation of Belgian endometriosis publications with a citation rate (CR) of 22.46 citations per single endometriosis publication. Sweden was located in the second position with a rate of 22.26 followed by Finland (CR 20.77), the USA (CR 20.13), Norway (CR 19.42), the Netherlands (CR 17.19), Canada (CR 16.69) and the UK (CR 15.94).

The country- and endometriosis-specific h-Index calculation (Fig. 2c) demonstrated a leading position of the USA with 103 endometriosis-related publications cited at least 103 times. The USA is followed by the UK with a country-specific h-Index (HI) of 57, Belgium (HI 49), Italy and Japan (HI both 47), France (HI 42) and Canada (HI 41). Again, countries from Asia (with the exception of Japan), Africa, Eastern Europe, or Latin America were characterized by very low citation rates.

### Cooperation articles

**Fig. 3:**
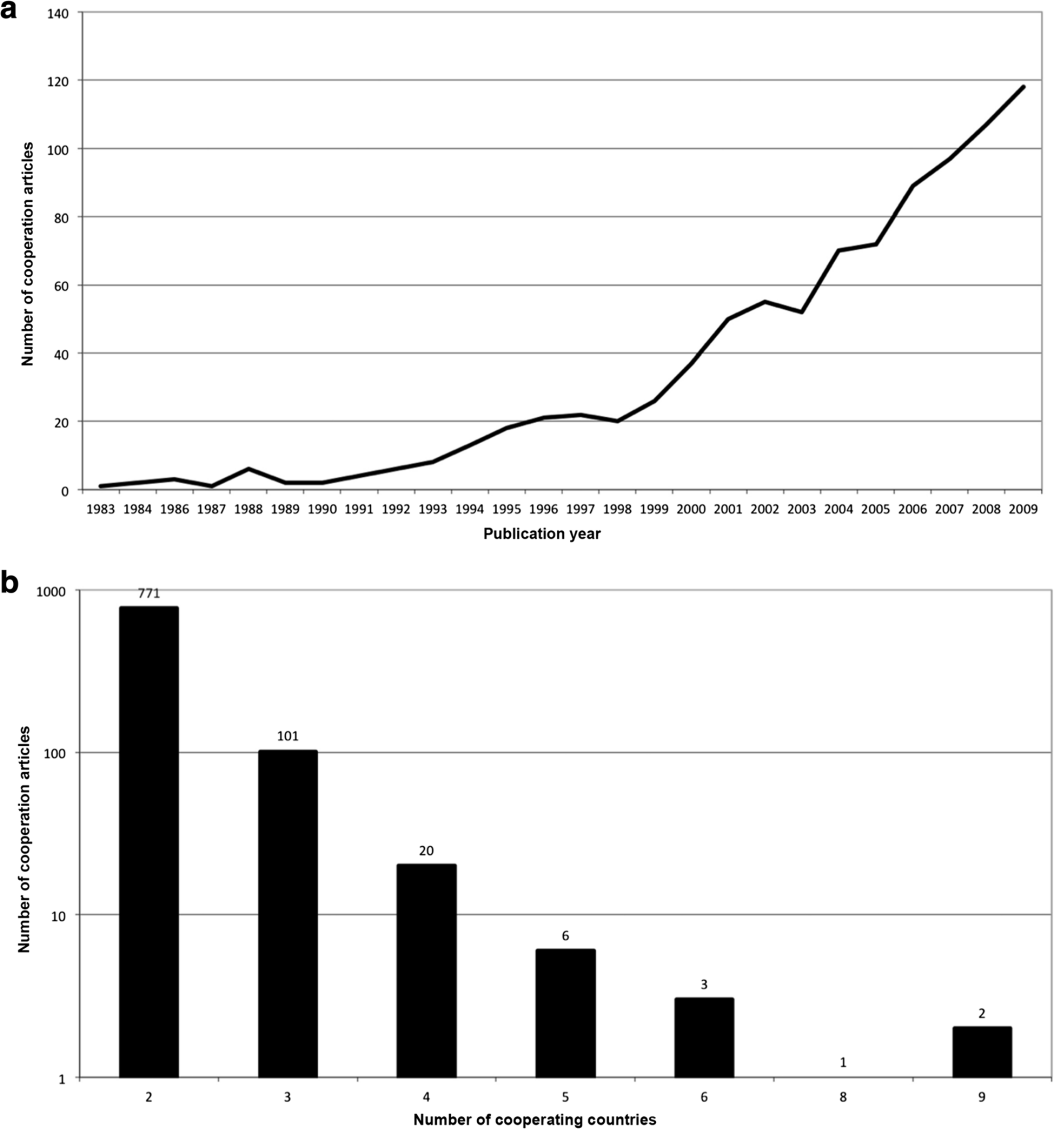
**a** International collaborations from 1983 to 2009. **b** Number of co-authors

In the period between 1900 and 2009, 902 of all publications were based on international collaborative efforts. This equaled a rather low percentage of 8.15 %. 771 of these items were issued by bilateral collaborations (85.5 %, Fig. 3a), followed by trilateral (101 publications) and collaborations between four countries (20 publications). Analysis of the chronological evolution indicated a strong increase in international collaborations in the past 10 years (Fig. 3b).

**Fig. 4:**
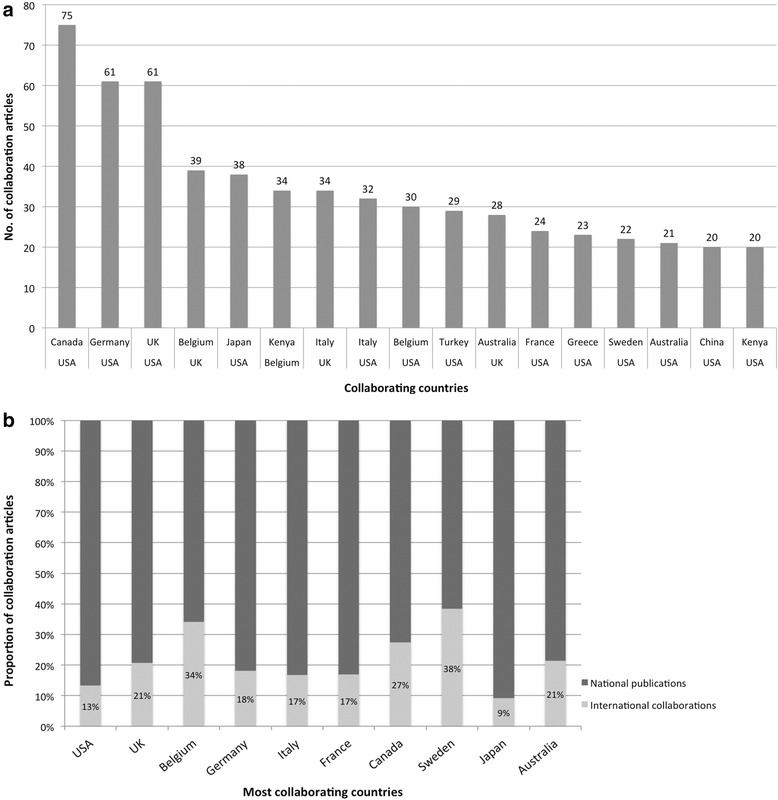
**a** Number of collaborative items published in a joint effort between two countries dedicated to research in the field of endometriosis. The figure depicts only cooperations of countries with more than 20 collaborative publications **b** Proportions of international collaborative efforts among all publications of the ten most proliferative countries in endometriosis research

Overall, the USA dominated the category of collaborative efforts in the field of endometriosis research; 493 publications could be attributed to international cooperations. It was followed by the UK (197 papers based on international collaborations out of 952 items in total), Germany (130 papers based on international collaborations out of 715 items in total), Belgium (129 papers based on international collaborations out of 378 items in total), and Italy (121 papers based on international collaborations out of 720 items in total). The USA established 75 bilateral collaborations with Canada, 61 with Germany and 61 with the UK. Other fruitful collaborations were identified between Belgium and the UK (39 items), Japan and the USA (38 items), Belgium and Kenya (34 items), Italy with the UK (34 items) and Italy with the USA (32 items, Fig. 4a). Among the ten countries having issued the most cooperation articles in the field, the percentage of publications based on international collaborative efforts were the lowest in Japan (9 %) and the USA (13 %). Belgium and Sweden constituted notable exceptions. These countries published more than a third of their total research output on endometriosis within the framework of international cooperations (Fig. 4b).

### Subject area analysis

**Fig. 5:**
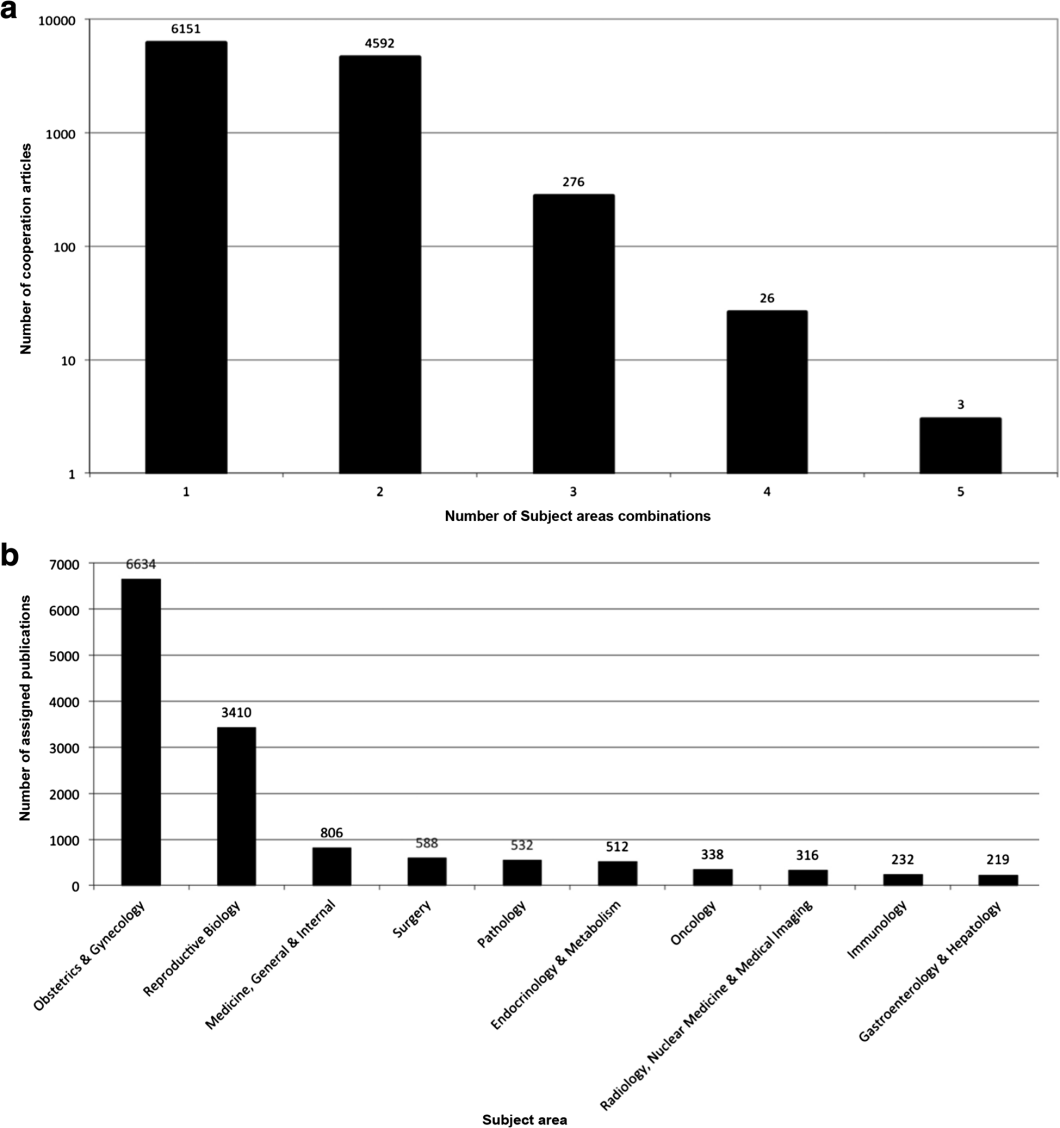
**a** Distribution of subject area assignments per endometriosis publication. **b** Most frequently assigned subject areas

Most endometriosis publications were assigned to one (6151) or two (4592) subject areas (Fig. 5a). We identified ‘Obstetrics and Gynecology’ as the most frequently applied subject category with 6634 assignments followed by ‘Reproductive Biology’ with 3410 assignments. Then, separated by a large gap, ‘Medicine, General & Internal’ was found (806 assignments), followed by ‘Surgery’ (588 assignments), *‘*Pathology’ (532 assignments) and *‘*Endocrinology & Metabolism’ (512 assignments) (Fig. 5b). The most frequent combination of two assigned subject areas was *‘Obstetrics & Gynecology’* with *‘Reproductive Biology’* attributable to 3014 publications. When focusing on differences in subject areas between the 15 most productive countries in endometriosis research, no large differences were found.

### Journal analysis

**Fig. 6:**
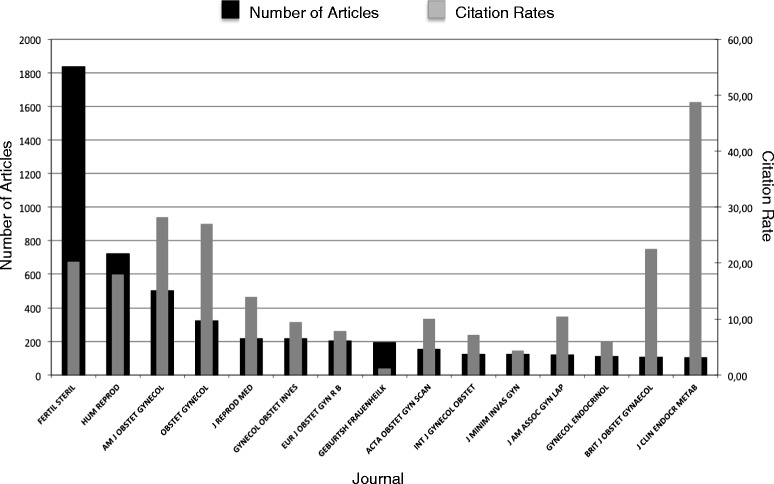
The 15 most prolific journals in endometriosis research with number of articles, number of citations and citation rate

The 15 most prolific journals in endometriosis research were identified and illustrated in Fig. 6. *“Fertility and Sterility”* was leading the field (1837 articles), followed by “Human Reproduction” (721 articles) and the “American Journal of Obstetrics and Gynecology” (501 articles). The highest citation rate was achieved by the *“Journal of Clinical Endocrinology and Metabolism”* (104 articles, CR = 48.66).

## Discussion

As elegantly described by Kvaskoff et al., the etiology of endometriosis remains poorly understood despite an estimated prevalence of 10 % in women [9]. The authors stress the fact that many unsolved questions still exist since endometriosis has been associated with an increased risk of several debilitating and chronic conditions, such as ovarian cancer, asthma, autoimmune, atopic and cardiovascular diseases [9]. Therefore, it is commonly accepted that a deeper understanding of these associations is needed as they may provide novel insights into the etiology or clinical implications of the condition [9]. Thus, research in this field needs a strong boost with new and profound funding programs to be initiated by agencies around the world. Since these endeavors require a sound underlying basis, we identified the need for an all-encompassing depiction of the existing global research landscape and conducted the first detailed analysis of worldwide endometriosis research. So far, only a few other OB/GYN topics including breast cancer [7, 27] and smoking and pregnancy [28] were subjected to a similar in-depth analysis using the NewQIS platform.

Global endometriosis research is dominated by a small number of scientific power players: These include the USA and the UK holding the leading positions in regards to publication quantity, country-specific citation numbers and h-Indices. Our data align with other studies evaluating the scientific productivity in the biomedical field. Groneberg-Kloft et al. analyzed over 5.5 million publications related to 22 organ systems and identified the USA in the top position regarding overall research output [29]. Comparing our results with other investigations in the areas of Internal Medicine or OB/GYN in particular leads to a similar picture: When scientific productivity was evaluated in regards to “tuberculosis”, bacterial meningitis”, “influenza” as well as “smoking in pregnancy” or “breast cancer”, the USA and the UK were once again dominating the field [28, 30–32]. This pattern might point to the fact that both countries dedicate major financial resources towards biomedical research creating a supportive scientific infrastructure, e.g. as indicated by the worldwide highest number of endometriosis research institutions (n: 1293) located in the USA. Also, both nations established numerous fruitful collaborations facilitating high quality research. Furthermore, we identified Belgium and Japan as prominent scientific forces within the endometriosis community. Japan’s strong interest in the field might be based on the fact that prevalences of endometriosis and associated clear-cell ovarian cancers are estimated to be the worldwide highest among Asian - and particularly Japanese - women [33, 34]. Therefore, the nation prioritizes related research with the aim to alleviate the associated disease burden for its inhabitants. For many years, Belgian scientists (e.g. at the University of Leuven) have been focusing on endometriosis research. Their efforts translated into numerous highly regarded publications as documented by the highest country-specific citation rate this nation received in our analysis. Concerning country-specific citation rates, we also found three Scandinavian countries (Sweden, Finland and Norway) among the five leading nations indicating the outstanding quality and high recognition of their publications. This finding might be linked to the vast data resources these countries established (e.g. The Swedish National Patient Register), which enable researchers to conduct meaningful and highly cited key studies in the field.

The need for an assessment of the worldwide endometriosis research architecture becomes even more pressing when the limited number of studies with a global perspective on endometriosis is compared with the fact that this enigmatic disease heavily affects female health in a global context - ranging from high income to low income countries and all ethnicities. In this respect, Nnoaham et al. stressed that little is known about the impact of endometriosis across the world [35]. Therefore, they initiated integrated multicenter studies to collect prospective, standardized, epidemiological data, to 1) examine the global impact of endometriosis and relative effect of risk-factors, and 2) develop a symptom-based diagnostic tool [35]. Their studies encompassed 19 hospitals in 13 countries [36, 37]. In one study, the authors assessed the impact of endometriosis on mental, physical and social well being and concluded that endometriosis impairs health-related quality of life and work productivity significantly across countries and ethnicities, yet women continue to experience diagnostic delays in primary care [37]. These findings stress the need for further multinational approaches, especially also with the participation of low income countries in which millions of women suffer from undiagnosed and insufficiently treated endometriosis. This claim is substantiated by our presented data: A closer look into the global endometriosis research architecture exemplifies that a large gap of research activity exists in a multitude of countries in which – under conservative estimations – tens of millions of affected women live, e.g. in China, India (BRIC countries – nations at a similar stage of newly advanced economic development - with established research infrastructures that could be instantly used for endometriosis research) and African countries. These numbers are mirrored by an extremely low number of endometriosis publications originating from these countries. This disparity verifies that research in this field is simply not a priority in developing or underdeveloped countries. Overall, research on the endometriosis in low resource countries is extremely scarce. Kyama et al. reflected on the problem of endometriosis in women living on the African continent and concluded in 2007 that research programs have to be developed and established to tackle the local unique challenges the condition creates [38]. The authors point out that rates of endometriosis are reported to be lower in African women compared with females in industrialized countries. They attributed this finding to an epidemiological under-estimation since the locally perceived need for accurate studies is low, to under-diagnosis due to limited awareness of both patients and doctors, restricted access to healthcare and surgical resources as well as a protective lifestyle (e.g. earlier childbearing, high parity). Our study further verifies the clear lack of research activity in countries of low-resources and reinforces Kyama’s call to action in particular. Hence, it would be key to extent the worldwide increasing collaborative efforts and involve these underserved nations with the aim to share data, resources and knowledge. In this context, we have to mention one exception, Kenya, showcasing successful international collaborative endeavors: This African nation has established 41 co-operations with other countries (e.g. with Belgium). As early as 1968, spontaneous endometriosis was reported in baboons [39, 40]. Since then, these monkeys, which are housed in the “Institute of Primate Research” in Nairobi (http://www.primateresearch.org), serve as an invaluable animal model in the field of endometriosis research leading to many milestone publications in the field [41–49].

Several methodological issues need to be discussed when interpreting the present data: 1) As an important strength of this study, the NewQIS platform constitutes an established protocol to assess basic and clinical sciences benchmarks in a standardized and verified way. So far, it was primarily used i.e. within the areas of public health [29, 50–53] and internal medicine [32, 54]. Now, it has been extended to OB/GYN; in this field, many diseases are treated that pose a major thread on female and pediatric well-being and have large public health implications. 2) Our platform is configurated to access the WoS database instead of PubMed. Therefore, differences in the overall number of identified endometriosis publications can be expected. This finding can be explained by the differing set of journals enlisted in both databases. However, we prefer to work with WoS as the primary database since it enables the user to integrate a concise citations analysis. This unique feature allowed us to perform valuable multifaceted semi-qualitative approaches, i.e. by calculating endometriosis-specific, country-specific h-indices or average citation rates. 3) A language bias constitutes a mentionable weakness of the presented NewQIS analysis: Clearly, the WoS - but also the PubMed - does not automatically index every Non-English scientific journal. By contrast, other databases such as Scopus include them. Since the majority of journals included in the WoS - and therefore analyzed in our search - are written in English an underrepresentation of non-English publications can be assumed. Especially in the area of public health and epidemiology, which are often focused on country-specific, national issues, there may be non-English endometriosis studies that are not traceable in the WoS. However, we consider this language bias as limited because data of outstanding interest and quality will be published in WoS-listed scientific journals and therefore included in our search.

## Conclusion

This is the first study to delineate a picture of the global research landscape of one of the most enigmatic diseases present in OB/GYN. In this area of medicine, the USA plays the leading role for the advancement of science. It is the country with the highest number of highly cited endometriosis publications and it shares the highest number of collaborations with other countries. It is also noteworthy that small Northern European countries such as Belgium, Finland and Sweden, seem to dedicate many resources in relation to their low numbers of inhabitants. An intensification of research efforts is needed to battle pressing challenges on a global scale such as the malignant transition of endometriosis or the development of novel non-invasive diagnostic tests and treatment approaches in order to improve disease management for millions of affected women in industrialized and developing countries. In this context, it should be a priority of supranational institutions such as the WHO or private funding organizations to provide the much needed monetary support.

## Additional file

Additional file 1: Most cited articles in endometriosis research. The table depicts the ten most cited articles in the area of endometriosis research are displayed including their title, publication year, country of origin, citation count and journal. (DOCX 113 kb)

## Abbreviations

CR: Citation report
DEMP: Density Equalizing Map Projections
HI: Hirsch-Index
NewQIS: New Quality and Quantity Indices in Science
OB/GYN: Obstetrics and Gynecology
UK: United Kingdom
USA: United States of America
WoS: Web of Science
