# Supplementary material for: Endometriosis and its global research architecture: an in-depth density-equalizing mapping analysis
Source: BMC Womens Health. 2016 Sep 21;16:64. doi: 10.1186/s12905-016-0336-0 (PMC5031306; doi:10.1186/s12905-016-0336-0)
Supplement: Additional file 1: — Most cited articles in endometriosis research. The table depicts the ten most cited articles in the area of endometriosis research are displayed including their title, publication year, country of origin, citation count and journal. (DOCX 113 kb) [file 12905_2016_336_MOESM1_ESM.docx]

Additional File 1. The table depicts the ten most cited articles in the area of endometriosis research are displayed including their title, publication year, country of origin, citation count and journal.

| **Title** | **Publication Year** | **Country** | **Citations** | **Journal** |
| --- | --- | --- | --- | --- |
| Peritoneal endometriosis due to the menstrual dissemination of endometrial tissue into the peritoneal cavity.  **Sampson JA** | 1927 | United States | 786 | AM J OBSTET GYNECOL |
| Revised American Society for Reproductive Medicine classification of endometriosis: 1996.  **No authors listed** | 1997 | United States | 423 | FERTIL STERIL |
| Ovarian steroid regulation of vascular endothelial growth factor in the human endometrium: implications for angiogenesis during the menstrual cycle and in the pathogenesis of endometriosis.  **Shifren JL et al.** | 1996 | United States | 384 | J CLIN ENDOCR METAB |
| Hysterectomy in the United States, 1988-1990.  **Wilcox LS et al.** | 1994 | United States | 365 | OBSTET GYNECOL |
| Medical Progress: Endometriosis.  **Olive DL and Schwartz LB** | 1993 | United States | 362 | N ENGL J MED |
| Retrograde menstruation in healthy women and in patients with endometriosis.  **Halme J et al.** | 1984 | United States | 344 | OBSTET GYNECOL |
| Suggestive evidence that pelvic endometriosis is a progressive disease, whereas deeply infiltrating endometriosis is associated with pelvic pain.  **Koninckx PR et al.** | 1991 | Belgium | 327 | FERTIL STERIL |
| Peritoneal endometriosis, ovarian endometriosis, and adenomyotic nodules of the rectovaginal septum are three different entities.  **Nisolle M and Donnez J** | 1997 | Belgium | 309 | FERTIL STERIL |
| Modulation of oestrogen receptor signalling by association with the activated dioxin receptor.  **Ohtake F et al.** | 2003 | Japan, France | 307 | Nature |
| Administration of Nasal Nafarelin as Compared with Oral Danazol for Endometriosis.  **Henzl MR et al.** | 1988 | United States | 304 | N ENGL J MED |
